# Predictors of response to prefabricated foot orthoses or rocker-sole footwear in individuals with first metatarsophalangeal joint osteoarthritis

**DOI:** 10.1186/s12891-017-1558-5

**Published:** 2017-05-12

**Authors:** Hylton B. Menz, Maria Auhl, Jade M. Tan, Pazit Levinger, Edward Roddy, Shannon E. Munteanu

**Affiliations:** 1 La Trobe Sport and Exercise Medicine Research Centre, School of Allied Health, La Trobe University, Melbourne, 3086 VIC Australia; 2 Discipline of Podiatry, School of Allied Health, La Trobe University, Melbourne, 3086 VIC Australia; 3 Arthritis Research UK Primary Care Centre, Research Institute for Primary Care and Health Sciences, Keele University, Staffordshire, ST5 5BG UK; 4 Institute of Sport, Exercise and Active Living, Victoria University, Melbourne, 8001 VIC Australia

**Keywords:** Osteoarthritis, Hallux rigidus, Shoes, Foot orthoses

## Abstract

**Background:**

Osteoarthritis of the first metatarsophalangeal joint (1st MTPJ OA) is a common and disabling condition commonly managed with footwear and orthotic interventions. The objective of this study was to identify factors associated with a successful treatment response in people with 1st MTPJ OA provided with prefabricated orthoses or rocker-sole footwear as part of a randomised clinical trial.

**Methods:**

People with 1st MTPJ OA (*n* = 88) who participated in a randomised trial were allocated to receive prefabricated foot orthoses (*n* = 47) or rocker-sole footwear (*n* = 41) and completed a baseline questionnaire including information on demographics, anthropometrics, general health, pain characteristics (including the Foot Health Status Questionnaire [FHSQ] and Foot Function Index [FFI]) and perceptions of the interventions, and a clinical assessment of foot posture, range of motion, radiographic severity and in-shoe plantar pressures. Adherence was documented using diaries. At 12 weeks, participants documented their perception of improvement on a 15-point scale. Those reporting at least moderate improvement on this scale were classified as ‘responders’.

**Results:**

There were 29 responders (62%) in the orthoses group and 16 responders (39%) in the rocker-sole group. In the orthoses group, responders had greater baseline pain severity while walking, a higher FFI difficulty score, and wore their orthoses more frequently. In the rocker-sole group, responders had a higher FFI stiffness score and greater radiographic severity. However, the accuracy of these variables in identifying responders in each group was modest (62 and 53%, respectively).

**Conclusion:**

The response to prefabricated orthoses or rocker-sole footwear in people with 1st MTPJ OA is related to measures of increased pain and disease severity. However, the overall classification accuracy associated with these factors is not sufficient for identifying individuals who are most likely to benefit from these interventions.

**Trial registration:**

Australian New Zealand Clinical Trials Registry: ACTRN12613001245785

## Background

Osteoarthritis (OA) of the first metatarsophalangeal joint (1st MTPJ) is the most common presentation of foot OA, affecting 7.8% of people aged over 50 years [1]. The condition is characterised by symptoms of joint pain and stiffness, formation of a dorsal exostosis, and progressive reduction in range of motion of the 1st MTPJ [2]. People affected by 1st MTPJ OA report associated locomotor disability [1] and decreased health-related quality of life [3]. Commonly recommended treatments for 1st MTPJ OA include physical therapies, anti-inflammatory medications, intra-articular injections, foot orthoses, footwear modifications and surgery [4]. However, few of these treatments have been rigorously evaluated [5].

In a recent trial, we found that prefabricated foot orthoses and rocker-sole footwear were similarly effective at reducing pain associated with 1st MTPJ OA [6]. However, the footwear group exhibited lower adherence, reported less global improvement in symptoms, and were more likely to experience adverse events compared to the orthoses group. Furthermore, although biomechanical analyses indicated that both interventions reduced peak pressure under the 1st MTPJ, they appeared to achieve this through different mechanisms, with the orthoses increasing pressure under the midfoot and lesser toes and the rocker-sole footwear decreasing pressure under the 2nd to 5th MTPJs [7].

The findings of our trial suggest that although both treatments appear to be effective, their acceptability and underlying mechanisms of action may differ. As such, there may be some value in identifying the characteristics of those who are most likely to respond favourably to each treatment, as this could potentially aid in the clinical decision making process. Previous studies of non-surgical treatments for musculoskeletal disorders (such as knee and hip OA and patellofemoral pain) have shown that a wide range of factors may influence clinical outcomes, including age, sex, body mass index, pain severity and duration, structural foot characteristics and range of motion [8–10]. However, no such studies have been undertaken for foot disorders in general, or 1st MTPJ OA specifically.

Therefore, the aim of this study was to identify factors associated with a successful treatment response in people with 1st MTPJ OA who were provided with prefabricated orthoses or rocker-sole footwear in the trial. We considered demographics, anthropometrics, general health, pain characteristics, perceptions of the interventions, structural foot characteristics, radiographic severity and in-shoe plantar pressures as possible predictors.

## Methods

The data presented in this paper are taken from a larger randomised trial (Australian New Zealand Clinical Trials Registry ID: ACTRN12613001245785). The La Trobe University Human Ethics Committee provided ethical approval (number 13-003) and all participants provided written informed consent prior to enrolment. The full trial protocol, including detailed inclusion and exclusion criteria, has been published previously [11]. Key components of the methods are reproduced in the following section.

### Participants

To be included in the study, participants had to have pain rated at least 20 mm on a 100 mm visual analogue scale (VAS) in the 1st MTPJ on most days for at least 12 weeks, and less than 64° of dorsiflexion range of motion of the 1st MTPJ [12]. Although radiographs were obtained to document the presence and severity of osteoarthritic changes (see ‘Questionnaires and clinical assessments’ section below), radiographic signs of OA were not an inclusion criterion. Key exclusion criteria included previous surgery on the 1st MTPJ, significant deformity of the 1st MTPJ including hallux valgus [13, 14], cognitive impairment (a score of <7 on the Short Portable Mental Status Questionnaire) [15], and older people with a history of recurrent falls, due to the short-term detrimental effects of rocker-sole shoes on balance [16].

### Questionnaires and clinical assessments

At baseline, participants completed a comprehensive questionnaire, clinical assessment and biomechanical assessment, which collected information on the following:

(i) demographics/anthropometrics: age, sex and body mass index (BMI);
(ii) general health/physical activity: the Short-Form-12 Version 2 questionnaire [17] and the Incidental and Planned Activity Questionnaire [18];
(iii) pain characteristics: pain duration (months), pain severity at rest and while walking, stiffness in the morning and later in the day (each via a 100 mm visual analog scale), the pain and function subscales of the the Foot Health Status Questionnaire (FHSQ) [19], and the pain, difficulty and stiffness subscales of the the Foot Function Index - Revised (Short Form) (FFI) [20];
(iv) foot posture and range of motion: the Foot Posture Index [21] and passive non-weightbearing dorsiflexion range of motion at the 1st MTPJ [22];
(v) radiographic severity of 1st MTPJ OA: using a standardised radiographic atlas [23], participants were divided into four radiographic severity groups (none = no radiographic evidence of OA, mild = at least one score of one for either osteophytes or joint space narrowing in either the dorso-plantar or lateral views, moderate = at least one score of two for either osteophytes or joint space narrowing in either the dorso-plantar or lateral views, or severe = at least one score of three for either osteophytes or joint space narrowing in either the dorsoplantar or lateral views) [2];
(vi) biomechanical effects of the interventions: in-shoe peak plantar pressures (kPa) were assessed with the in-shoe Pedar^®^ system (Novel GmbH, Munich, Germany) when participants were wearing their own shoes and the intervention, and the percentage change in peak pressure beneath the 1st MTPJ was documented;
(vii) credibility and expectancy: the Credibility/Expectancy Questionnaire (CEQ) [24], which consists consists of six items: three related to credibility and three related to expectancy. For each item, participants were asked to rate the credibility of the treatment and their expectations on a 9-point Likert scale;
(viii) footwear perceptions: participants allocated to the rocker-sole footwear group were asked to report their perceptions of shoe attractiveness (to self and to others), comfort, ease of donning and doffing, fit and heaviness, using questions selected from the Monitor Orthopaedic Shoes questionnaire [25], scored on a 100 mm visual analog scale. The selected questions were: (i) Please mark on the following line how attractive you think the shoes are (with the anchors “extremely unattractive” and “extremely attractive”), (ii) Please mark on the following line how attractive you think other people would think the shoes are (with the anchors “extremely unattractive” and “extremely attractive”), (iii) Please mark on the following line how comfortable you think the shoes are (with the anchors “extremely uncomfortable” and “extremely comfortable)”, (iv) Please mark on the following line how well you think the shoes fit you (using the anchors “poorest fit possible” and “best fit possible”), (v) Please indicate how easy it is for you to don the shoes on and off (using the anchors “most difficult as possible” and “as easy as imaginable”) and (vi) Please indicate how heavy the shoes are (using the anchors “extremely light” and “extremely heavy”);
(ix) adherence: total hours the intervention was worn over the 12 week follow-up period.

### Interventions

**Fig. 1:**
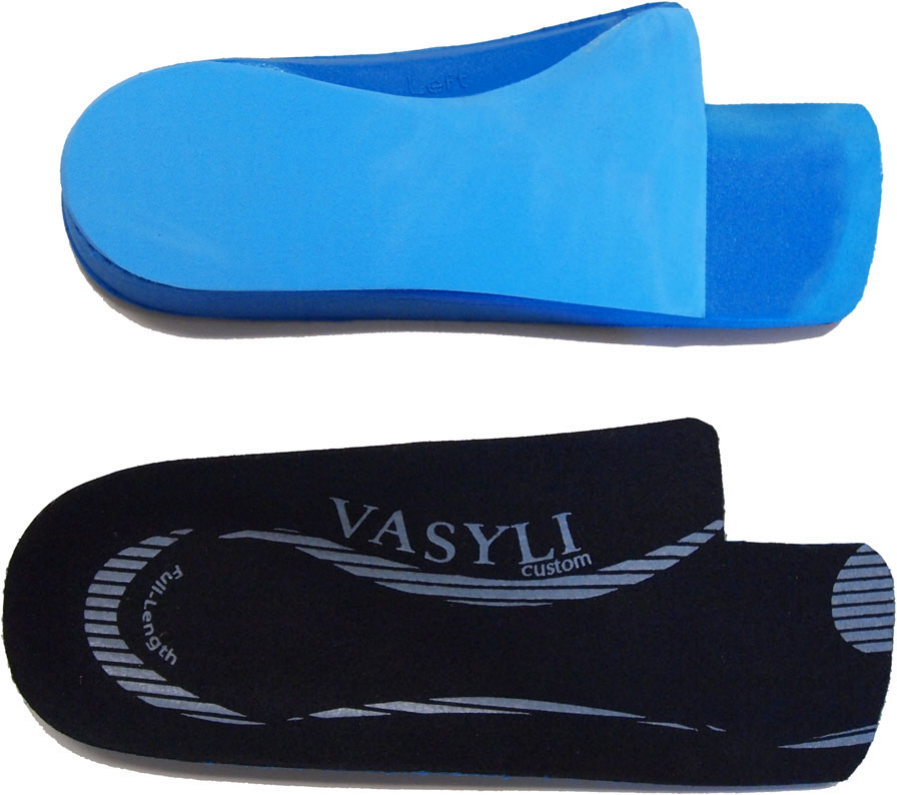
Prefabricated foot orthoses used in the trial. *Top*: plantar surface of *left foot* orthosis. *Bottom*: dorsal surface of *right foot* orthosis. Figure from Menz et al. [8]

The prefabricated foot orthoses group received a pair of foot orthoses (Vasyli Customs Medium Density, Vasyli Medical™, Queensland, Australia) that were modified using a similar approach to that described by Welsh et al. [26], involving the addition of a cut-out section beneath the first metatarsal and trimming the distal edge to the level of the 2nd to 5th toe sulci (Fig. 1). In participants with pronated feet (a Foot Posture Index [FPI] score of >7 [27]), full length 4-degree medial (varus) wedges were applied to orthoses until there was a reduction in the FPI score of at least 2 points [26]. This was required for 2 participants.

**Fig. 2:**
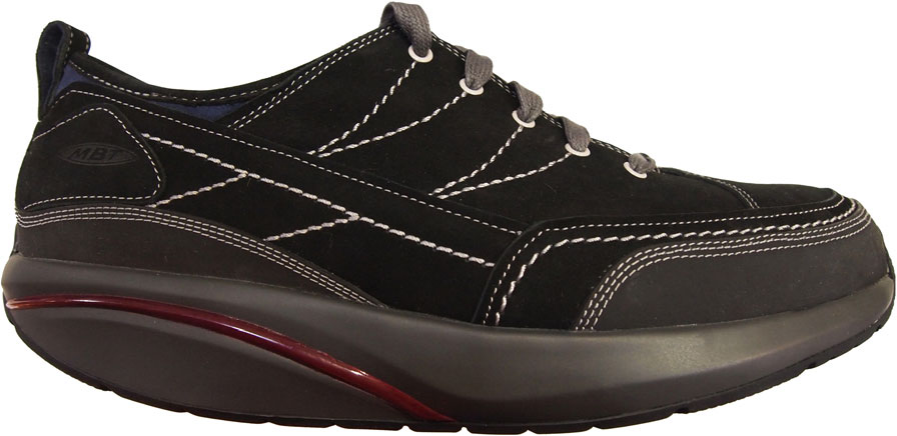
MBT^®^ Matwa footwear used in the trial. Figure from Menz et al. [8]

The rocker-sole footwear group were provided with a pair of MBT^®^ shoes (Masai Barefoot Technology, Switzerland) (Fig. 2) [28]. Four participants received the Mahuta model and 42 received the Matwa model, as the Mahuta was discontinued shortly after study commencement. However, both models had the same sole curvature and only differed in relation to the aesthetics of the upper.

### Definition of responders and non-responders

At the 12 week follow-up, participants documented their perception of overall improvement on a 15-point scale using the descriptors “a very great deal better” (score = 7), “a great deal better” (score = 6), “a good deal better” (score = 5), “moderately better” (score = 4), “somewhat better” (score = 3), “a little better” (score = 2), “about the same, hardly any better at all” (score = 1), “no change” (score = 0), “about the same, hardly any worse at all” (score = −1), “a little worse (score = −2), “somewhat worse” (score = −3), “moderately worse” (score = −4), “a good deal worse” (score = −5), “a great deal worse” (score = −6) and “a very great deal worse” (score = −7) [29]. Those reporting a score of 4 or above (i.e. at least “moderately better”) were defined as responders, while those reporting a score of less than four were defined as non-responders.

### Statistical analysis

Statistical analysis was undertaken using SPSS version 22.0 (IBM Corp, NY, USA). The most symptomatic foot was selected as the index foot for all analyses, or in the case of equivalent symptoms in both feet, the right foot was selected. All data were explored for normality using the skewness statistic (−1 to 1). Comparisons between responders and non-responders within each intervention group were compared using chi-square tests (for categorical data), independent samples t-tests (for normally distributed continuous data) or Mann-Whitney U tests (for skewed continuous data). Variables found to be significantly different between responders and non-responders (at *p* < 0.05) were then entered into a discriminant function analyses using the ‘enter’ method. The relative importance of each variable in discriminating between responders and non-responders was determined using standardised canonical discriminant function coefficients. After deriving each discriminant function, cross-validation was carried out using the jack-knife procedure, and the accuracy of the model in identifying responders in each group was expressed as a percentage [30].

## Results

### Participants

A total of 102 participants were randomised into the main study [6]. Complete baseline and 12 week follow-up data for this analysis were available from 88 participants (47 in the orthoses group and 41 in the rocker-sole footwear group). Of these, 29 (62%) were classified as responders in the orthoses group and 16 (39%) were classified as responders in the rocker-sole footwear group.

### Differences between responders and non-responders in the orthoses group

In the orthoses group, there were no differences between responders and non-responders in relation to demographics/anthropometrics, general health, foot posture and range of motion, radiographic severity, the biomechanical effects of the interventions or treatment credibility and expectancy. However, responders exhibited greater baseline pain severity while walking, a higher FFI difficulty score, and wore their orthoses more frequently than non-responders (see Table 1).

**Table 1:** Characteristics of non-responders and responders in the orthoses group (*n* = 47)

	Non-responders (*n* = 18)	Responders (*n* = 29)
Demographics/anthropometrics		
Age, years–mean (SD)	59.0 (11.3)	57.1 (10.0)
Females–*n* (%)	8 (44)	18 (62)
Body mass index, kg/m^2^–mean (SD)	27.9 (5.1)	29.6 (4.72)
General health/physical activity		
SF 12 physical component score–mean (SD) ^a^	43.2 (12.1)	45.2 (10.2)
SF 12 mental component score–mean (SD) ^a^	57.1 (9.5)	54.4 (6.5)
Physical activity, total hours per week–mean (SD)	20.9 (10.5)	17.1 (17.2)
Pain characteristics		
Pain duration, months–median (IQR)	33 (101)	30 (59)
Pain severity at rest–mean (SD) ^b^	3.1 (3.0)	3.4 (2.3)
Pain severity while walking–mean (SD) ^b^	3.8 (2.3)	5.2 (2.1)^*^
Stiffness in the morning–mean (SD) ^b^	2.4 (2.4)	3.6 (2.8)
Stiffness later in the day–mean (SD) ^b^	2.3 (2.7)	3.9 (2.7)
FHSQ pain–mean (SD) ^a^	61.5 (23.4)	53.5 (17.6)
FHSQ function–mean (SD) ^a^	77.1 (18.6)	66.2 (24.8)
FFI pain–mean (SD) ^a^	34.8 (15.6)	44.8 (17.9)
FFI difficulty–mean (SD) ^a^	26.9 (20.2)	43.9 (26.3)^*^
FFI stiffness–mean (SD) ^a^	28.1 (18.7)	36.7 (20.8)
Pain Catastrophising Scale–mean (SD) ^b^	8.2 (7.8)	10.0 (8.9)
Foot posture and range of motion		
Foot posture index–mean (SD)	2.9 (2.7)	2.9 (2.4)
1st MTPJ range of motion (°)–mean (SD)	41.2 (12.6)	40.3 (11.7)
Radiographic severity		
None–*n* (%)	0 (0)	0 (0)
Mild–*n* (%)	3 (17)	12 (41)
Moderate–*n* (%)	7 (39)	6 (21)
Severe–*n* (%)	8 (44)	11 (38)
Biomechanical effects of intervention		
1st MTPJ peak pressure: own shoes, kPa–median (IQR)	188.8 (100.0)	150.0 (60.0)
1st MTPJ peak pressure: intervention, kPa–median (IQR)	161.3 (68.8)	125.0 (70.0)
% reduction in 1st MTPJ peak pressure–median (IQR)	14.0 (24.7)	13.6 (16.3)
Credibility and Expectancy Questionnaire		
Credibility–mean (SD)	19.6 (2.4)	21.2 (3.2)
Expectancy–mean (SD)	17.3 (3.4)	16.9 (4.1)
Total hours intervention worn during study–mean (SD)	332 (202)	518 (245)^*^

*SD* standard deviation, *IQR* interquartile range

^a^lower scores indicate worse health status/greater impairment

^b^higher scores indicate worse health status/greater impairment

^*^ *p* < 0.05

### Differences between responders and non-responders in the rocker-sole footwear group

In the rocker-sole footwear group, there were no differences between responders and non-responders in relation to demographics/anthropometrics, general health, foot posture and range of motion, the biomechanical effects of the interventions, credibility and expectancy, adherence or footwear perceptions. However, responders had a higher FFI stiffness score and greater radiographic severity than non-responders (see Table 2).

**Table 2:** Characteristics of non-responders and responders in the rocker-sole footwear group (*n* = 41)

	Non-responders (*n* = 25)	Responders (*n* = 16)
Demographics/anthropometrics		
Age, years–mean (SD)	56.9 (8.4)	55.9 (12.4)
Females–*n* (%)	14 (56)	11 (69)
Body mass index, kg/m^2^–mean (SD)	28.4 (4.1)	28.3 (4.9)
General health/physical activity		
SF 12 physical component score–mean (SD) ^a^	45.8 (10.2)	45.4 (9.8)
SF 12 mental component score–mean (SD) ^a^	52.2 (8.1)	51.7 (10.8)
Physical activity, total hours per week–mean (SD)	14.5 (11.0)	16.8 (10.1)
Pain characteristics		
Pain duration, months–median (IQR)	36 (108)	24 (75)
Pain severity at rest–mean (SD) ^b^	3.4 (2.7)	3.5 (2.3)
Pain severity while walking–mean (SD) ^b^	4.7 (2.2)	5.1 (2.1)
Stiffness in the morning–mean (SD) ^b^	3.9 (2.9)	4.1 (1.6)
Stiffness later in the day–mean (SD) ^b^	3.7 (2.8)	3.7 (2.2)
FHSQ pain–mean (SD) ^a^	52.6 (19.7)	45.9 (17.9)
FHSQ function–mean (SD) ^a^	70.8 (23.4)	62.5 (28.0)
FFI pain–mean (SD) ^a^	41.1 (16.6)	47.1 (18.3)
FFI difficulty–mean (SD) ^a^	37.2 (25.3)	45.9 (23.3)
FFI stiffness–mean (SD) ^a^	31.8 (22.9)	44.4 (16.7)^*^
Pain Catastrophising Scale–mean (SD) ^b^	9.2 (6.9)	10.6 (7.9)
Foot posture and range of motion		
Foot posture index–mean (SD)	3.5 (2.0)	3.6 (2.2)
1st MTPJ range of motion (°)–mean (SD)	41.4 (13.4)	38.5 (13.8)
Radiographic severity		
None–*n* (%)	1 (4)	3 (20)
Mild–*n* (%)	3 (13)	2 (13)
Moderate–*n* (%)	16 (70)	2 (13)
Severe–*n* (%)	3 (13)	8 (53)^*^
Biomechanical effects of intervention		
1st MTPJ peak pressure: own shoes, kPa–mean (SD)	167.5 (62.5)	158.3 (40.6)
1st MTPJ peak pressure: intervention, kPa–mean (SD)	133.2 (49.1)	137.3 (36.8)
% reduction in 1st MTPJ peak pressure	19.5 (18.0)	11.2 (19.8)
Credibility and Expectancy Questionnaire		
Credibility–mean (SD)	21.0 (3.6)	21.3 (3.3)
Expectancy–mean (SD)	17.1 (5.3)	19.1 (4.4)
Footwear perceptions (100 mm visual analog scales)		
Shoe attractiveness (to self)–mean (SD)	40.1 (20.4)	50.7 (26.3)
Shoe attractiveness (to others)–mean (SD)	38.4 (20.7)	48.7 (21.8)
Shoe comfort–mean (SD)	61.6 (26.6)	64.3 (18.5)
Ease of donning and doffing shoes–mean (SD)	76.6 (21.1)	79.6 (16.5)
Perception of shoe fit–median (IQR)	82.0 (21.0)	77.5 (16.0)
Perception of shoe heaviness–mean (SD)	46.1 (25.2)	44.7 (18.7)
Total hours intervention worn during study–mean (SD)	278 (200)	320 (179)

*SD* standard deviation, *IQR* interquartile range

^a^lower scores indicate worse health status/greater impairment

^b^higher scores indicate worse health status/greater impairment

^*^ *p* < 0.05

### Discriminant function analysis

Results of the discriminant function analysis are shown in Table 3. In the orthoses group, the discriminant function model was significant (*p* = 0.012), and the combination of three predictor variables identified responders with 73% accuracy (62% following validation). In the footwear group, the discriminant function model did not reach statistical significance (*p* = 0.175), and the combination of two predictor variables identified responders with 63% accuracy (53% following validation).

**Table 3:** Discriminant function analysis

	Orthoses group (*n* = 47)	Rocker-sole footwear group (*n* = 41)
Wilk’s lambda, chi-square, *p*	λ = 0.77, *χ* ^2^ = 10.9, *p* = 0.012	λ = 0.91, *χ* ^2^ = 3.5, *p* = 0.175
Predictor variable (discriminant function coefficient)	Total hours intervention worn during study (0.681)	Foot Function Index stiffness (1.00)
	Pain severity while walking (0.468)	Radiographic severity (0.287)
	Foot Function Index difficulty (0.361)	
% of cases correctly classified	73.3	63.2
% of cases correctly classified following validation	62.2	52.6

## Discussion

The objective of this study was to identify factors associated with a successful treatment response in people with first metatarsophalangeal joint osteoarthritis (1st MTPJ OA) who were provided with prefabricated orthoses or rocker-sole footwear as part of a recently completed randomised clinical trial [6]. To do this, we administered a comprehensive baseline questionnaire and clinical assessment (including measures of demographics, anthropometrics, general health, pain characteristics, perceptions of the interventions, foot posture, range of motion, radiographic severity and in-shoe plantar pressures) and compared these characteristics between those who did and did not respond to each of the interventions at 12 weeks of follow up. We found that several measures of pain and disease severity differed between responders and non-responders. However, the ability of these measures to accurately discriminate between the groups was only modest.

In the orthoses group, responders had greater baseline pain severity while walking and a higher FFI difficulty score, while in the rocker-sole group, responders had a higher FFI stiffness score and greater radiographic severity. Taken together, these findings suggest that both interventions may be more effective in those with more severe symptoms or more advanced disease. These observations are consistent with previous studies of OA affecting the knee or hip. Greater baseline pain severity has been shown to predict better outcomes of rehabilitation for knee [8] and hip OA [9], while greater radiographic severity is associated with better outcomes following knee and hip replacement surgery [31–34]. These findings also add some support to the proposed biomechanical effects of these interventions, as the variables found to differ between responders and non-responders related primarily to functional limitation of the 1st MTPJ (eg: pain while walking rather than at rest, and FFI items indicative of difficulty with ambulation).

The only other significant predictor of response we found was intervention adherence, with responders in the orthoses group wearing their intervention for a significantly greater number of hours throughout the course of the trial than non-responders. No such difference was found in the rocker-sole footwear group, although the overall adherence in this group was substantially lower, possibly due to aesthetic concerns about the footwear and workplace attire constraints. Although no studies have explored the role of adherence in predicting outcomes of footwear-related interventions in foot OA, it has previously been shown that greater adherence with prescribed footwear is associated with a lower rate of foot ulcer recurrence in people with diabetic peripheral neuropathy [35, 36]. However, given that adherence was documented throughout the trial, it is also possible that adherence increased as a consequence of reduction in pain in some individuals, rather than being a cause of pain reduction.

The current biomechanical theory underpinning the use of mechanical interventions for 1st MTPJ OA would suggest that those with greater range of motion may be more likely to benefit from orthoses, as the removal of material beneath the 1st MTPJ would facilitate first ray plantarflexion during propulsion, thereby allowing the proximal phalanx to rotate on the first metatarsal head to achieve sufficient 1st MTPJ dorsiflexion [37]. In contrast, those with less range of motion may be more likely to benefit from the rocker-sole footwear, as the curved sole reduces the need for 1st MTPJ dorsiflexion during propulsion [38]. However, neither the range of motion of the 1st MTPJ, nor the changes in 1st MTPJ plantar pressure associated with the interventions differed between responders and non-responders in either group. This may be because the available 1st MTPJ range of motion measured when non-weightbearing is only moderately associated with the range used during gait [39], and that vertical pressures may not provide a valid indicator of compressive forces within the 1st MTPJ.

This study has a number of limitations. Firstly, we were unable to use the OMERACT-OARSI criteria [40] to define responders, as these criteria are based on Western Ontario McMaster Universities Osteoarthritis (WOMAC) index scores in people with hip or knee OA. In the absence of any established criteria to define responders in foot OA, we consider our cut-off score (at least “moderately better” on a 15-point scale) to be justifiable, but acknowledge that this is essentially arbitrary. Secondly, although we included a broad array of potential predictor variables, we acknowledge that other measures may have provided additional insights. For example, direct measurement of 1st MTPJ kinematics and kinetics using a marker-based optoelectronic gait analysis system may have provided greater insight into changes in joint function, however this was not possible in our trial as it would have required permanent modification of the participants’ footwear to accommodate the markers. A more objective measure of adherence (such as using a wearable sensor [41]) would also have been useful. Thirdly, we were unable to include a non-treatment control arm in this trial, so we cannot exclude the contribution of contextual effects to the apparent improvements in both treatment groups. Finally, although we identified several factors which differed between responders and non-responders, the discriminant function analysis indicated that the classification accuracy was modest and unlikely to be sufficient for identifying patients who are most likely to respond in a clinical setting.

## Conclusion

People with 1st MTPJ OA who responded favourably to prefabricated orthoses had more baseline pain and difficulty walking, and wore their orthoses more frequently during the trial, while those who responded favourably to rocker-sole footwear had greater baseline joint stiffness and radiographic OA severity. However, the overall classification accuracy associated with these factors was modest and is unlikely to be sufficient for identifying responders in a clinical setting. Further study is therefore required to identify factors that predict a positive treatment response to treatments for 1st MTPJ OA.

## Abbreviations

1st MTPJ: First metatarsophalangeal joint
BMI: Body mass index
CEQ: Credibility/Expectancy Questionnaire
FFI: Foot Function Index
FHSQ: Foot Health Status Questionnaire
FPI: Foot Posture Index
OA: Osteoarthritis
VAS: Visual analogue scale
WOMAC: Western Ontario McMaster Universities Osteoarthritis index
